# Electrochemical Solvent Cointercalation into Graphite in Propylene Carbonate-Based Electrolytes: A Chronopotentiometric Characterization

**DOI:** 10.1155/2018/9231857

**Published:** 2018-03-06

**Authors:** Hee-Youb Song, Soon-Ki Jeong

**Affiliations:** Department of Chemical Engineering, Soonchunhyang University, Asan, Chungnam 336-745, Republic of Korea

## Abstract

Interfacial reactions strongly influence the performance of lithium-ion batteries, with the main interfacial reaction between graphite and propylene carbonate- (PC-) based electrolytes corresponding to solvent cointercalation. Herein, the redox reactions of solvated lithium ions occurring at the graphite interface in 1 M·LiClO_4_/PC were probed by chronopotentiometry, in situ atomic force microscopy (AFM), and in situ Raman spectroscopy. The obtained results revealed that high coulombic efficiency (97.5%) can be achieved at high current density, additionally showing the strong influence of charge capacity on the above redox reactions. Moreover, AFM imaging indicated the occurrence of solvent cointercalation during the first reduction, as reflected by the presence of hills and blisters on the basal plane of highly oriented pyrolytic graphite subjected to the above process.

## 1. Introduction

Graphite is extensively used for the fabrication of lithium-ion batteries (LIBs) [1–4] due to being capable of electrochemically intercalating lithium ions into graphene layers and thus enabling electrical energy storage during charging. However, the above intercalation reaction is strongly affected by the nature of the utilized electrolytes, which comprise lithium ions, counter anions, and organic solvents [5]. In electrolyte solutions, the interaction between lithium ions and organic solvents leads to the solvation of the former [6–8], which is undesired and should be inhibited due to solvated lithium ions being intercalated into graphite at more positive potentials than nonsolvated ones [9–12]. In addition, the continuous cointercalation of coordinated solvent leads to graphite exfoliation.

Despite its remarkable ionic conductivity at low temperature (melting point = −49°C), propylene carbonate (PC), widely used as a main organic solvent in primary lithium batteries, has not been applied to LIBs due to undergoing ceaseless cointercalation into graphite during the first charging [13–16]. PC cointercalation is well known to occur at ∼1 V versus Li^+^/Li, hindering the lowering of the electrode potential to values corresponding to lithium intercalation (0.25–0.0 V versus Li^+^/Li) and thus inducing graphite exfoliation prior to lithium intercalation during the first charging. Thus, solvent cointercalation significantly degrades the performance of LIBs and should be suppressed to allow reversible intercalation/deintercalation of lithium ions at graphite-negative electrodes.

The effective suppression of solvent cointercalation at graphite-negative electrode requires a deep understanding of numerous factors of influence. Commonly, the irreversible reduction of solvated lithium ions at ∼1 V is the main interfacial reaction in PC-based electrolyte solutions, indicating the importance of understanding the redox behavior of PC-solvated lithium ions. Herein, we probed the above behavior by chronopotentiometry, characterizing the potential change upon application of negative/positive currents and investigated the effects of current density on the redox reactions of solvated lithium ions. Moreover, in situ atomic force microscopy (AFM) and in situ Raman spectroscopy were used to clarify certain aspects of solvent cointercalation during the first reduction reaction.

## 2. Materials and Methods

### 2.1. Preparation of Electrode Materials and Electrolyte Solution

Natural graphite powder (NG-7, Kansai Coke, and Chemicals Co.) was used as an active electrode material for chronopotentiometry. A composite working electrode was prepared by coating copper foil (Nilaco Co.) with a 9 : 1 (w/w) mixture of NG-7 and poly(vinylidene difluoride) and drying it at 80°C in a vacuum oven (Yamato Scientific Co., DNE401) for 12 h. Highly oriented pyrolytic graphite (HOPG; Advanced Ceramics, ZYH grade, mosaic spread = 3.5 ± 1.5°) was used as a model electrode for in situ AFM imaging, which was carried out for freshly cleaved HOPG surfaces. Lithium foil (Honjo Metal Co.) was used as reference and counter electrodes in all electrochemical measurements, and a 1 M solution of LiClO_4_ in PC (Kishida Chemical Co., battery grade) was used as an electrolyte.

### 2.2. Chronopotentiometry

Chronopotentiometric measurements were performed using a battery test system (Hokuto Denko, HJ101SM6), with 2032 coin cells tested at various C-rates (1 C = 372 mA·g^−1^) to understand the effect of current density on the redox behavior of solvated lithium ions.

### 2.3. In Situ AFM

The basal plane of HOPG was imaged in contact mode using a pyramidal silicon nitride tip (OLYMPUS Co., OMCL-TR800PSA) in 1 M·LiClO_4_/PC. AFM images (5 *μ*m × 5 *μ*m) were automatically captured at each potential during cyclic voltammetry (CV) scans between 3.0 and 0.0 V (scan rate = 2 mV·s^−1^) using an AFM imaging system (Molecular Imaging, PicoSPM^®^) equipped with a potentiostat (Molecular Imaging, PicoStat). All AFM characterizations were performed at room temperature in an argon-filled glove box (Miwa, MDB-1B + MM3-P60S, dew point < −70°C).

### 2.4. In Situ Raman Spectroscopy

An electrochemical (quartz) cell for in situ Raman spectroscopy was assembled in an argon-filled glove box, sealed, and removed from the glove box into ambient atmosphere. The 514.5 nm line of an argon-ion laser was scattered on HOPG during the first reduction by applying a constant current of 1 C. Raman spectra were collected using a triple monochromator (Jobin-Yvon, T64000) equipped with a multichannel charge-coupled device detector.

## 3. Results and Discussion

### 3.1. Effect of Current Density on Solvent Cointercalation

**Figure 1:**
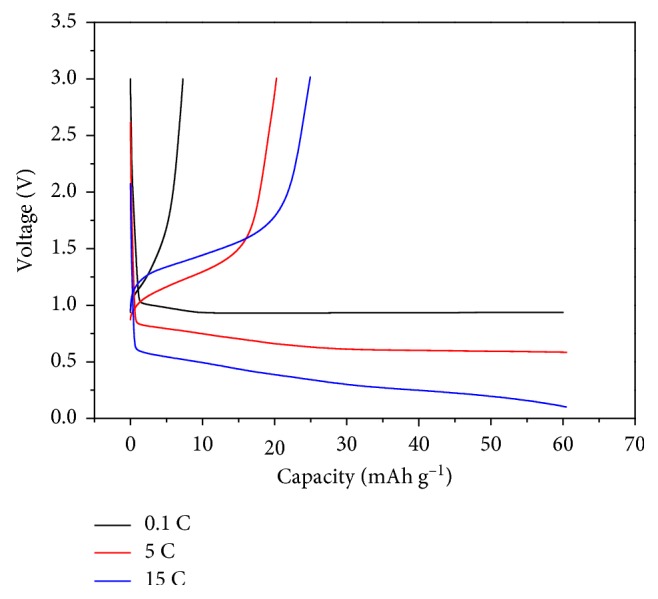
Voltage profiles of NG-7 at various C-rates in 1 M·LiClO_4_/PC, with the cell charged to 60 mAh·g^−1^ at each cycle.

Figure 1 shows voltage profiles recorded at various C-rates in 1 M·LiClO_4_/PC. During these measurements, the graphite-negative electrode was charged to 60 mAh·g^−1^ and instantly discharged without any rest time, providing insights into the redox behavior of solvated lithium ions above the lithium intercalation potential, with a different discharge capacity observed in each cycle reflecting the oxidation of solvated lithium ions. In general, solvent cointercalation (corresponding to the reduction of solvated lithium ions) takes place at ∼1 V in PC-based electrolytes [13–16]. However, in this case, the solvent cointercalation potential decreased, and the discharge capacity increased as the current density increased from 0.1 to 5 or 15 C, indicating the occurrence of reversible redox reactions of solvated lithium ions at graphite and additionally showing that the oxidation of solvated lithium ions can be controlled by choosing an appropriate current density.

**Figure 2:**
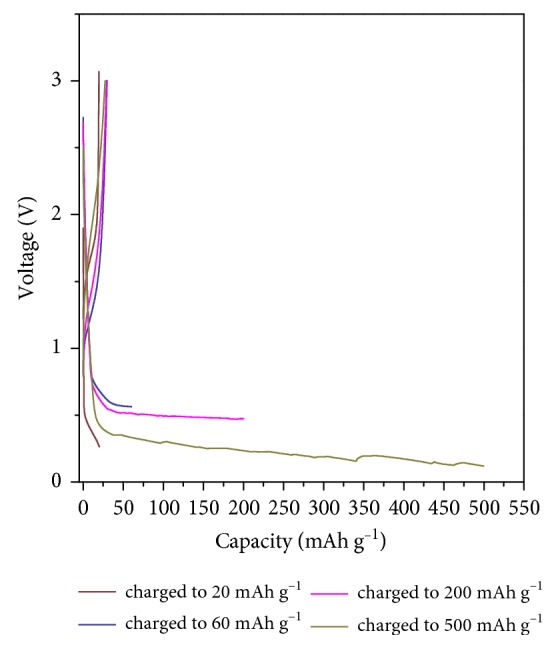
Voltage profiles of NG-7 at various charge capacities and a C-rate of 5 C in 1 M·LiClO_4_/PC.

For further experiments, we selected a C-rate of 5 C and investigated the discharge capacity of fabricated cells to understand the effect of charge capacity on the redox reactions of solvated lithium ions. Notably, no significant increase of discharge capacity (∼20 mAh·g^−1^) was observed as the charge capacity increased from 20 to 500 mAh·g^−1^ (Figure 2). Moreover, coulombic efficiency increased with decreasing charge capacity, indicating that the redox reactions of solvated lithium ions could be controlled by varying both current density and charge capacity.

**Figure 3:**
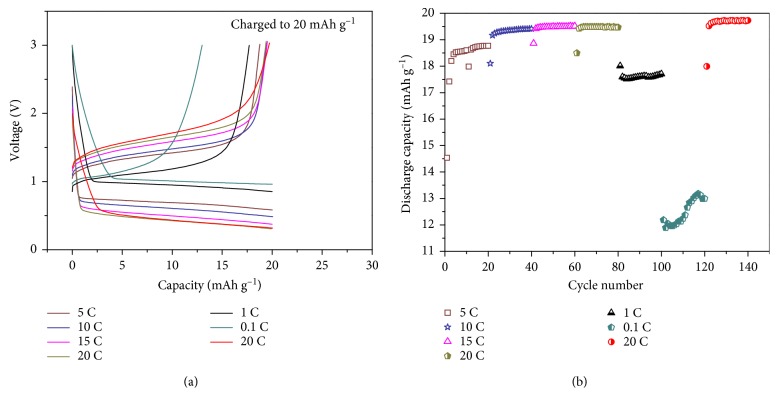
(a) Voltage profiles and (b) cycling performance of NG-7 at various C-rates in 1 M·LiClO_4_/PC, with the cell charged to 20 mAh·g^−1^.

Figure 3(a) shows charge and discharge capacities obtained for a cell charged to 20 mAh·g^−1^ above the lithium intercalation potential at various current densities. The corresponding coulombic efficiency was estimated as 65% at 0.1 C, increasing to ≥97.5% at 10, 15, and 20 C and thus indicating that most solvated lithium ions cointercalated into graphite were oxidized at the above high current densities during discharge. Furthermore, the discharge capacity was sustained after 140 cycles (Figure 3(b)). Thus, the redox reactions of solvated lithium ions at graphite in PC-based electrolytes were demonstrated to be dependent on current density and charge capacity.

### 3.2. In Situ AFM and Raman Investigation of HOPG during the First Reduction

**Figure 4:**
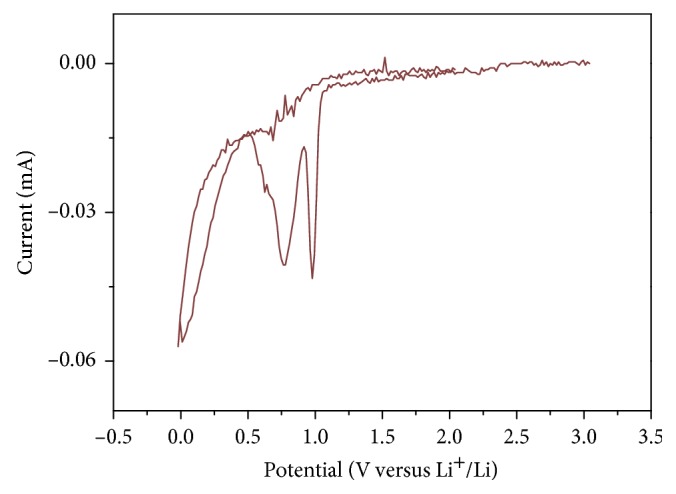
Cyclic voltammogram of HOPG during the first cycle in 1 M·LiClO_4_/PC recorded at a scan rate of 2 mV·s^−1^.

**Figure 5:**
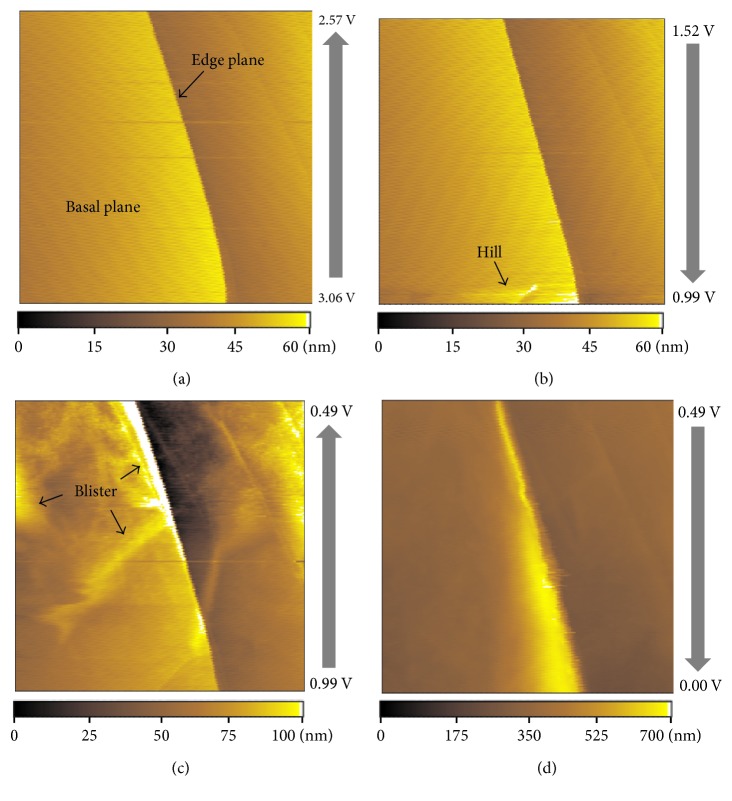
In situ AFM images of the HOPG basal plane (5 *µ*m × 5 *µ*m) obtained at (a) 3.06–2.57, (b) 1.52–0.99, (c) 0.99–0.49, and (d) 0.49–0.0 V during the first reduction in 1 M·LiClO_4_/PC.

Morphological changes of the HOPG basal plane were investigated by in situ AFM to obtain further insights into solvent cointercalation in PC-based electrolytes.  Figure 4 shows a cyclic voltammogram of HOPG in 1 M·LiClO_4_/PC, revealing the presence of three reduction peaks at potentials below 1.0 V during the first cycle. Prior to reduction, HOPG exhibited a flat surface comprising basal and edge planes (Figure 5(a)), with significant morphological changes observed after reduction; that is, hill-like structures were detected on the HOPG basal plane at ∼1 V (Figure 5(b)). Similarly, Inaba et al. observed related structures on the surface of graphite after cointercalation of solvated lithium ions, ascribing the formation of blisters to the decomposition of solvated lithium ions within graphite [11, 13]. Accordingly, the reduction peak at ∼1 V was attributed to solvent cointercalation into graphite, with blistering at decreased cell potentials (Figure 5(c)) explained as mentioned above. In addition, the height of the HOPG basal plane increased in a potential range of 0.49–0.0 V (Figure 5(d)).

**Figure 6:**
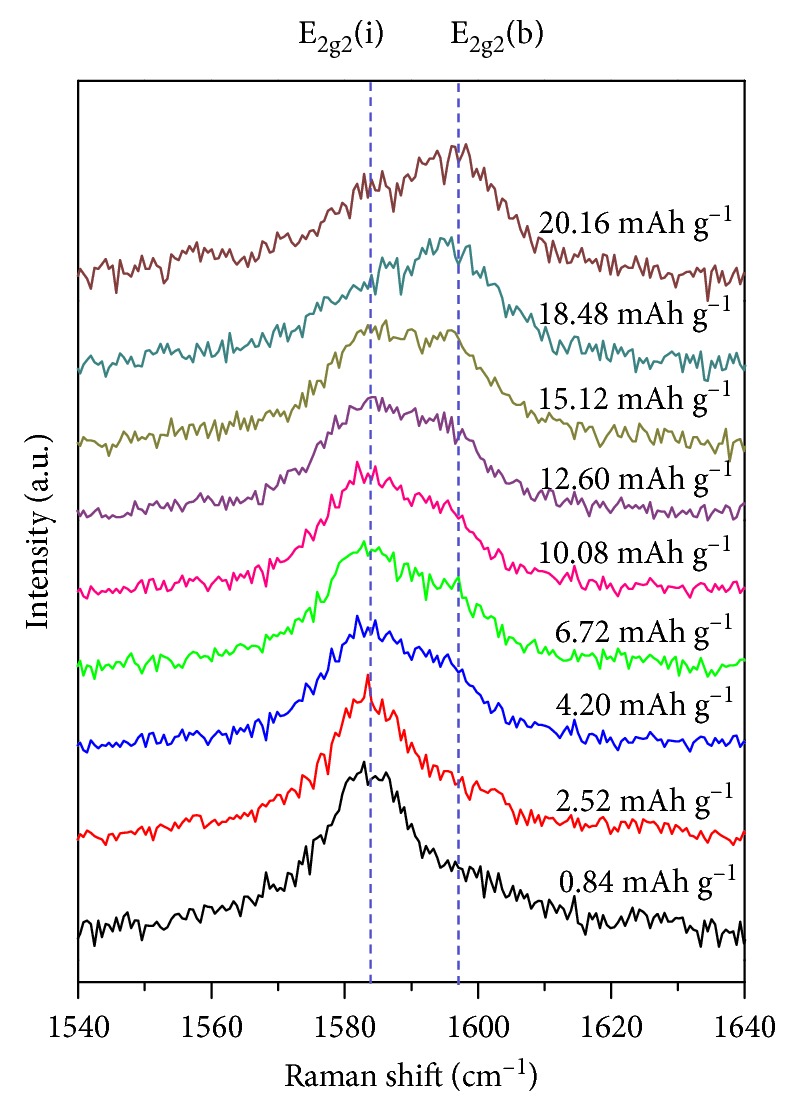
In situ Raman spectra of HOPG recorded during the first reduction in constant-current mode (1 C) in 1 M·LiClO_4_/PC. The electrochemical cell was charged to ∼20 mAh·g^−1^.

We also employed in situ Raman spectroscopy to clarify the nature of the intercalate formed during the first reduction of graphite in constant-current mode in a PC-based electrolyte. In this case, the electrode potential did not drop to the lithium intercalation potential while the graphite electrode was charged to 20 mAh·g^−1^ in the same manner as shown in Figure 3(a). However, both E_2g2_ (interior) and E_2g2_(boundary) bands at 1583 and 1597 cm^−1^, respectively, were observed during the first reduction reaction, indicating the absence and presence of intercalates within graphite layers, respectively (Figure 6). Moreover, the first reduction increased the intensity of the latter band and decreased that of the former, indicating that continuous cointercalation of PC-solvated lithium ions into graphite occurred at a potential higher than that of lithium intercalation. Based on the insights provided by in situ AFM and in situ Raman analysis, the results of chronopotentiometric characterization were attributed to the redox reactions of solvated lithium ions in the PC-based electrolyte.

## 4. Conclusions

Herein, we investigated the redox reactions of solvated lithium ions at graphite in 1 M·LiClO_4_/PC by chronopotentiometry, in situ AFM, and in situ Raman spectroscopy, revealing that these reactions were reversible at high current density and thus highlighting the key role of the latter parameter. Moreover, the discharge capacity related to the oxidation of solvated lithium ions increased with increasing current density, and improved coulombic efficiency was observed at high current densities of 10, 15, and 20 C in the case of charging to 20 mAh·g^−1^. AFM imaging revealed the appearance of hills and blisters on the otherwise smooth electrode surface during the first reduction, indicating the occurrence of solvent cointercalation and decomposition at graphite. Thus, the redox reactions of solvated lithium ions were shown to be the main interfacial reactions taking place at the graphite-negative electrode, with their control being possible by appropriate variation of current density and charge capacity.
